# The efficacy and safety of Tuina for diabetic gastroparesis: A protocol for systematic review and meta-analysis

**DOI:** 10.1097/MD.0000000000027964

**Published:** 2021-11-24

**Authors:** Huixin Yan, Yun An, Tao Zhang, Jiangna Zhao, Juntao Yan

**Affiliations:** Department of Tuina, Yueyang Hospital of Integrated Traditional Chinese and Western Medicine, Shanghai University of Traditional Chinese Medicine, Shanghai, P.R. China.

**Keywords:** diabetic gastroparesis, meta-analysis, systematic review, Tuina

## Abstract

**Background:**

Diabetic gastroparesis (DGP) is one of the most common complication of diabetes. At present, despite the increasing number of clinical researches of treatment with Tuina have been reported, there are no systematic reviews of Tuina therapy for DGP. Therefore, it is of great significance to evaluate its safety and efficacy as a complementary and alternative therapy.

**Methods and analysis:**

Randomized controlled trials (RCTs) of Tuina therapy in the treatment of DGP will be retrieved from PubMed, Embase, Web of Science, Cochrane Library, China National Knowledge Infrastructure (CNKI), Wan Fang Database (Wan Fang), Chinese Biomedical Literature Database (CBM), VIP Database for Chinese Technical Periodicals (VIP), Medline, and Clinical Trial Register (CTR). We will consider articles published in English or Chinese between database initiation and October 2021. Research selection, data extraction, risk of bias assessment, and meta-analyses will be independently completed by 2 researchers. Our researchers will use Revman 5.3 software, provided by the Cochrane Collaborative Network for conducting systematic reviews and meta-analysis.

**Results:**

This systematic review provides a high-quality synthesis to assess the effectiveness and safety of Tuina therapy for treating DGP.

**Conclusion:**

The conclusions of our study will provide evidence to determine whether Tuina therapy is an effective and safe intervention for patients with DGP.

**Systematic review registration:**

: INPLASY2021110019

## 1 Introduction

Diabetic gastroparesis (DGP) is a common chronic refractory gastrointestinal complication of diabetes, and it is increasingly recognized as a significant health issue.^[1,2]^ This nomenclature was created by Kassander in 1958.^[3]^ DGP is characterized by delayed gastric emptying in the absence of any mechanical obstruction with associated upper gastrointestinal symptoms.^[4,5]^ Patients living with DGP typically experience gastrointestinal symptoms, including early satiety, postprandial fullness, nausea, vomiting, abdominal distension or pain, and weight loss.^[6,7]^ Among them, abdominal pain is increasingly recognized as one of the most common symptoms of this disease.^[8]^ Diabetes is the most frequent cause of gastroparesis, approximately one-third of diabetic patients suffer from DGP.^[9,10]^ Consequently, as the number of diabetes continues to grow, the number of patients with DGP is increasing as well.^[10]^ It has been reported that DGP is much more common in type 1 diabetes mellitus (T1DM) than in type 2 diabetes mellitus (T2DM).^[11]^ Compared with normal individuals, the risk of DGP for T2DM is more than 7-fold higher, while T1DM have a 30-fold higher risk of DGP.^[12]^ A large survey of Chinese diabetic patients found that there were 70.5% patients presented with gastrointestinal symptoms.^[13]^ In the current guidelines, the strategy to manage DGP essentially focus on blood glucose control, nutritional optimization, and supportive nutrition.^[14]^ Modalities of treatments for DGP involve medications, nutritional support, gastric electrical stimulation (GES), injections of botulinum toxin, endoscopes, surgical interventions, and traditional Chinese medicine (TCM) therapy. These methods can be used individually or in combination.^[15]^ However, these current treatments have significant limitations, so there is no single method that is universally accepted as the gold standard for DGP.^[16]^ For most patients with refractory DGP, invasive and expensive treatment options are frequently adopted. But these methods can cause mental and psychological symptoms including mental distress, anxiety, depression, and not all patients are likely to benefit from these treatments.^[15]^ It has been estimated that about 2% to 5% of DGP patients require multiple hospitalizations for severe symptoms.^[15]^ Therefore, many patients are urgently seeking safer and more effective alternative therapies. In clinical practice, Tuina may deliver a safe and comfortable choice.

Tuina, as one of the unique complementary and alternative therapeutics in TCM, can be performed using techniques and standardized movements at specific sites on the body to achieve the purpose of modifying or curing disease. According to the TCM meridian theory, stimulating specific meridians and acupoints can regulate the functions of viscera and meridians, so as to cure diseases and enhance health. In recent years, Tuina has been extensively used for the treatment of patients with DGP, and it has received more and more public attention as both its green and safety positive effects are being concerned. However, many patients still do not benefit from this treatment owing to the lack of rigorous evidence-based medical evidence. Therefore, this study aimed to evaluate the effectiveness and safety of Tuina therapy, explored the feasibility of generalizing this method to the clinic and providing a treatment reference for clinicians.

## 2 Methods and analysis

### 2.1 Study registration

This study will be performed by adhering to the guidelines of the preferred reporting items for systematic review and meta-analysis protocols (PRISMA-P) 2015^[17]^. The study was registered at the International Platform of Registered Systematic Review and Meta-Analysis Protocols (INPLASY) on November 6, 2021 (registration number INPLASY2021110019).

### 2.2 Inclusion criteria

#### 2.2.1 Types of participants

Studies will include patients diagnosed with DGP, and presenting 2 or more of the following symptoms: abdominal bloating, pain, belching, nausea, vomiting, loss of appetite, diarrhea, and constipation; The outcomes of related examinations (gastric emptying examination, B-ultrasound, endoscopy, and so forth) were delayed gastric emptying and/or retention of food residues. No limitations of age, gender, location, ethnicity, race, profession, educational background, and economic status were imposed. However, studies on patients with serious underlying diseases such as cardiovascular disease, liver and kidney dysfunction, and mental illness will be excluded.

#### 2.2.2 Types of interventions

Interventions of the experimental group will include Tuina (such as chiropractic, viscera massage, acupoint massage) or combined with WM. However, the types of Tuina techniques will not be restricted. The control group received treatment with Western medication, Chinese herbal medication, placebo, acupuncture, acupoint injection, and so on, or even with no treatment, will be included. In short, Tuina should be the only difference between the experimental group and the control group.

#### 2.2.3 Types of studies

This review will include randomized controlled trials (RCTs) on Tuina for DGP published in Chinese and English language. The studies involving non-RCTs, animal experiments, retrospective studies, case series, cohort studies, case--control studies, and review articles will be excluded.

#### 2.2.4 Types of outcomes

The main outcome of this review is clinical total effective rate. The additional outcomes include FPG, 2 hour PPG, TCM symptom score, recurrence rate of clinical signs, adverse reactions, etc.

### 2.3 Data sources and search methods

#### 2.3.1 Electronic searches

We will search several databases, including PubMed, Embase, Web of Science, Cochrane Library, China National Knowledge Infrastructure (CNKI), Wan Fang Database (Wan Fang), Chinese Biomedical Literature Database (CBM), VIP Database for Chinese Technical Periodicals (VIP), Medline, and Clinical Trial Register (CTR) for eligible RCTs up to October 2021. According to the “PICOS” (patients, intervention, comparison, outcome, and study design) criteria, we use the following search terms: Diabetic gastroparesis, DGP, Tuina, massage, manipulation, Randomized controlled trial, RCT, etc. The search strategy for PubMed is presented in Table 1. The specific search strategy was adjusted according to the characteristics of different databases.

**Table 1:** Search strategy for the PubMed database.

Number	Search items
1	Tuina
2	Massage
3	Manipulation
4	Zone Therapy
5	Manual therapy
6	Massotherapy
7	Acupressure
8	1 OR 2-7
9	Diabetic gastroparesis
10	Diabetic gastric paralyze
11	Mellitus gastroparesis
12	DGP
13	9 OR 10-12
14	Randomized controlled trial
15	Controlled clinical trial
16	Clinical trial
17	Randomly
18	Placebo
19	Trial
20	RCT
21	14 OR 15-20
22	8 And 13 And 21

#### 2.3.2 Searching for other resources

We will also use related search terms to obtain in the Chinese Clinical Registry and the International Clinical Trials Registry Platform (ICTRP). In addition, we will manually search for the original literature to find possible related trials, and tried to obtain gray literature from other sources.

### 2.4 Data selection

**Figure 1:**
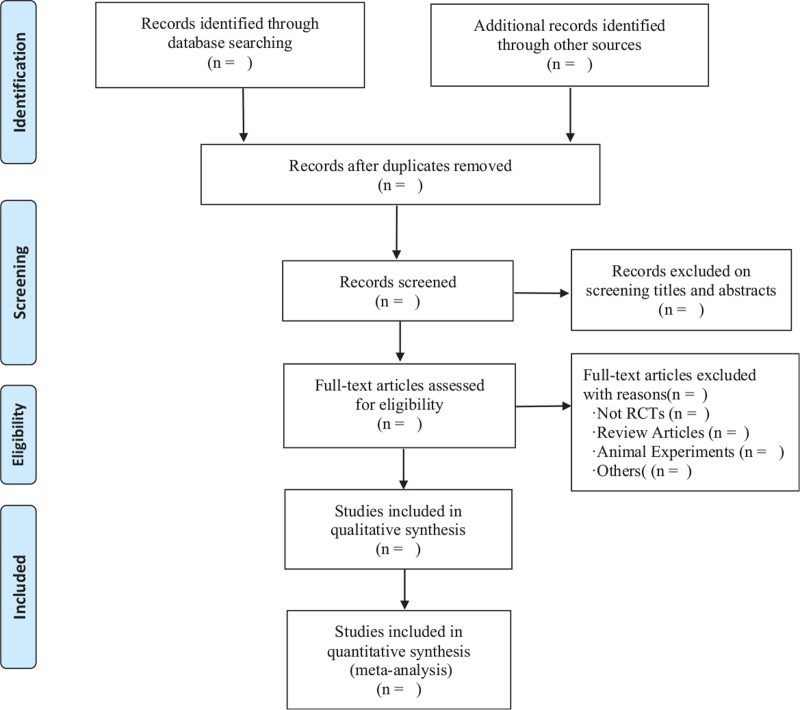
Flow diagram of the study selection process.

Before the start of the research, all researchers will carefully study the Preferred Reporting Items for Systematic Reviews and Meta-Analyses (PRISMA) and Cochrane Handbook for Systematic Reviews of Interventions (CHSRI). First of all, 2 researchers will import all records into NoteExpress V3.0 software or Endnote X9.1 software for collation, and duplicate literature will be deleted by the software. Then, 2 authors will screen the literature that meet the inclusion criteria by reading the title, abstract, and keywords. Finally, we will read the full text of the remaining literature that meet the standards and conduct an in-depth review to further determine whether to include or exclude them. If there are any differences, the researchers will discuss, negotiate, and resolve. The flow chart of literature search and screening process will be shown in Fig. 1.

### 2.5 Data extraction and analysis

According to the inclusion criteria and exclusion criteria, our study team built a data extraction tablet before data extraction. The basic information in final inclusion of the studies we need to extract will include: first author, publication year, trial location, study design, randomization method, sample size, age, gender, intervention method, treatment time, treatment frequency, and other information. In the process of collecting information, any differences will be resolved through team negotiation. If significant information is missing, we will try to contact the authors of the primary studies using e-mail.

### 2.6 Risk of bias assessment

Two researchers will use the Cochrane Collaborative tool to independently assess the risk of bias in all final inclusion of trials. Random sequence generation, allocation concealment, blinding of participants and personnel, blindness of outcome assessments, incomplete outcome data, selective reporting, and other bias will be evaluated as low, high, or ambiguous risk in each study. The results of the bias assessment risk will be carefully checked by other researchers, and any disagreement will be finalized after further discussion by all investigators.

### 2.7 Data synthesis and analysis

Review Manager software (Revman, Version 5.3 for Windows) will be used to perform the meta-analysis. The Chi-squared test and *I*^2^ statistic will be used to assess the heterogeneity of literature according to the values of *P* and *I*^2^. If the homogeneity is low (*P* > .1; *I*^2^ < 50%), the fixed-effect model will be used for the meta-analysis; otherwise, the random effect model will be selected. However, when the heterogeneity is particularly high, we will first try to find the cause of its occurrence, and then decide the final meta-analysis model to choose.

### 2.8 Assessment of reporting biases

If the number of included studies exceeds 10 trials, we will use Revman 5.3 software to generate funnel plots to report publication bias. When discovering asymmetrical funnel plot, we will try to analyze the possible causes with the Egger method.

### 2.9 Subgroup analysis

When there was substantial heterogeneity in the included studies, the subgroup analysis will be conducted according to factors such as condition of disease, course of disease, treatment sites, intervention frequency, age, gender, region, year, sample size, and other factors.

### 2.10 Sensitivity analysis

Sensitivity analysis is also one of the indispensable methods to deal with heterogeneity. The sensitivity analysis, we will conduct, is to explore the impact of trial bias risk on preliminary results. If there is significant statistical heterogeneity, sensitivity analysis will exclude low-quality studies, and repeat meta-analysis based on sample size and insufficient data to assess quality and robustness.

### 2.11 Grading the quality of evidence

According to the Grading of Recommendations Assessment, Development and Evaluation (GRADE) system, ^[18]^ 2 investigators will independently assess the quality of evidence. On the basis of the 5 rating standards of limitation, inaccuracy, inconsistency, indirectness, and publication bias, the quality of evidence will be divided into 4 levels such as high, medium, low, and very low.

### 2.12 Ethics and dissemination

Ethical approval was not required for this protocol, as no animal experiments and clinical trials were involved. In addition, we will publish the results in peer-reviewed journals.

## 3 Discussion

DGP is a diabetic complication mainly characterized by abdominal distension, abdominal pain, belching, nausea, vomiting, and loss of appetite. TCM believes that DGP belongs to the category of “distention and fullness,” “vomiting,” and “abdominal pain.” Western medication remains a mainstay of DGP treatment, and nutritional support and other treatment modalities usually as an adjuvant therapy. Massage therapy is a typical external treatment of TCM; it has been increasingly attractive for patients due to its definite curative effect and low side effects.

Through dredging of the meridians, adjustment of Zang-fu function, Tuina therapy can improve the symptoms of digestive system. From modern medicine point of view, Tuina can improve microcirculation, regulate insulin secretion, repair the function of the autonomic nervous system, and promote metabolism, so as to for achieving the purpose of the clinical symptoms of DGP treatment. ^[19]^ Previous studies have reported that Tuina can benefit patients with DGP. However, there has been no systematic review and meta-analysis on the therapeutic effect and safety of DGP by Tuina. Thus, this study is the first to systematically investigate this issue, in order to provide high-quality evidence and guidance to clinicians. Meanwhile, we hope to promote further research and development and promotion of Tuina.

## Author contributions

**Conceptualization:** Juntao Yan.

**Data curation:** Yun An, Tao Zhang, Jiangna Zhao.

**Formal analysis:** Huixin Yan.

**Funding acquisition:** Juntao Yan.

**Project administration:** Juntao Yan.

**Validation:** Huixin Yan, Tao Zhang.

**Writing – original draft:** Huixin Yan, Yun An, Tao Zhang, Jiangna Zhao.

**Writing – review & editing:** Huixin Yan, Yun An, Tao Zhang.
